# Integrative Analysis of Vaginal Microorganisms and Serum Metabolomics in Rats With Estrous Cycle Disorder Induced by Long-Term Heat Exposure Based on 16S rDNA Gene Sequencing and LC/MS-Based Metabolomics

**DOI:** 10.3389/fcimb.2021.595716

**Published:** 2021-03-02

**Authors:** GaiHong An, Yu Zhang, LiJun Fan, JiaJun Chen, MengFan Wei, Chao Li, XueWei Chen, Li Zhang, DanFeng Yang, Jing Wang

**Affiliations:** ^1^Department of Operational Medicine, Tianjin Institute of Environmental and Operational Medicine, Tianjin, China; ^2^Department of Endocrinology, Tianjin Central Hospital of Gynecology and Obstetrics, Tianjin, China

**Keywords:** estrous cycle disorder, long-term heat exposure, neurotransmitter, serum metabolomics, vaginal microbiota

## Abstract

Long term heat exposure (HE) leads to estrous cycle disorder (ECD) in female rats and damages reproductive function. However, the regulation mechanism of vaginal microorganisms and serum metabolomics remains unclear. This study aimed to explore the effects of microbes on the vaginal secretions of rats with ECD and describe the serum metabolomics characteristics and their relationship with vaginal microorganisms. The alterations in the serum levels of neurotransmitters were used to verify the possible regulatory pathways. The relative abundance, composition, and colony interaction network of microorganisms in the vaginal secretions of rats with ECD changed significantly. The metabolomics analysis identified 22 potential biomarkers in the serum including lipid metabolism, amino acid metabolism, and mammalian target of rapamycin and gonadotropin-releasing hormone (GnRH) signaling pathways. Further, 52 pairs of vaginal microbiota–serum metabolites correlations (21 positive and 31 negative) were determined. The abundance of *Gardnerella* correlated positively with the metabolite L-arginine concentration and negatively with the oleic acid concentration. Further, a negative correlation was found between the abundance of *Pseudomonas* and the L-arginine concentration and between the metabolite benzoic acid concentration and the abundance of *Adlercreutzia*. These four bacteria–metabolite pairs had a direct or indirect relationship with the estrous cycle and reproduction. The glutamine, glutamate, and dopamine levels were significantly uncontrolled. The former two were closely related to GnRH signaling pathways involved in the development and regulation of HE-induced ECD in rats. Serum neurotransmitters partly reflected the regulatory effect of vaginal microorganisms on the host of HE-induced ECD, and glutamatergic neurotransmitters might be closely related to the alteration in vaginal microorganisms. These findings might help comprehend the mechanism of HE-induced ECD and propose a new intervention based on vaginal microorganisms.

## Introduction

Women have a low tolerance to a high-temperature environment (Marsh and Jenkins, 2002). Long-term heat exposure (HE) has noticeable adverse effects on female mammals. Emerging evidence has confirmed that HE can lead to female endocrine disorders and ovarian dysfunction (Dickson et al., 2018), affect rat ovarian cell proliferation and apoptosis, induce ovarian hormone over secretion (Sirotkin, 2010), destroy hormone balance (Li et al., 2017), increase sensitivity of granulosa cells to apoptosis (Luo et al., 2016), and so on. Many studies reported the damage of HE-induced female reproductive function, yet its mechanism has not been fully elucidated. As global temperature rises, the scope of an environment of high temperature has expanded, and the period of high temperature is prolonged. The study on the mechanism of the effect of HE on female reproduction is both necessary and indispensable.

Microecological and metabolism alterations are closely related to the occurrence and regulation of diseases (Ziklo et al., 2018). 16S ribosomal deoxyribonucleic acid (16S rDNA) gene sequencing exists in all bacterial genomes, and hence it is highly conserved and specific. Metabonomics can use biological samples (urine, feces, blood, tissue, etc.) to reflect the body’s metabolic response to external stimuli (Sun et al., 2019). At present, the sequencing of the 16S rDNA gene with metabolomics is a standard method based on the analysis of gut microbiota and metabolism used to study the potential adverse effects of diseases and external stimuli (Zhao et al., 2018). Recently, it has been reported that HE affected microbiota diversity in some animals. For example, it caused the increase of Proteobacteria and the decrease of Bacteroides in pig feces (Xiong et al., 2020) and induced significant changes in the variety of duodenum, jejunum, ileum flora in Shaoxing duck (Tian et al., 2020) and cow fecal microbiota (Chen et al., 2018). Neonatal microbial colonization was affected *via* affecting maternal microbial transmission in late gestational HE in a pig model (He et al., 2020). Besides, significant changes were seen in energy metabolism in HE (including phosphorus, alkaline phosphatase, total protein, et al) stress response indices, hormones and immune factors were disorder in pigs (Xiong et al., 2020). The changes of serum indicators concentrations were monitored in crossbred cattle, including lactate dehydrogenase, aspartate aminotransferase, alanine aminotransferase, alkaline phosphatase and protein, urea, creatinine and triglyceride (Yadav et al., 2016), and the increase of the levels of plasma cortisol and cytokines in dairy cows (Chen et al., 2018).

Vaginal microorganisms are complex microbiota with different numbers and relative proportions (Martin, 2012). Under physiological conditions, vaginal microbiota reflects the metabolic, local, and hormonal conditions of women regarding quantity and quality (Borges et al., 2014). Vaginal microbiota can stimulate the host to release metabolites, which can not only affect the immune system but also enter the bloodstream and affect the circulatory system (Dasari et al., 2016; Tooli et al., 2019). Studies have shown that vaginal microbiota is closely related to reproductive function, and the functional balance between vaginal microbiota and host significantly affects reproductive function (Kaur et al., 2020). Sex hormones strongly affect the overall structure and function of vaginal microbiota (Hillier, 2011; Kaur et al., 2020). In general, vaginal microbiota is relatively balanced and stable, whereas genetic, ethnic, environmental, and behavioral factors can cause an imbalance of vaginal microbiota (De Seta et al., 2014). This imbalance may also be induced by alterations in sex hormones during HE (Bradley et al., 2018; An et al., 2020). Once vaginal microbiota is out of balance, it is sensitive to external factors and diseases, such as bacterial vaginosis (BV) (Kaambo et al., 2018) and human papillomavirus infection (Chorna et al., 2020). The alterations in vaginal secretions have a significant effect on the health, reproduction, and immunity of the body (Ziklo et al., 2018). The impacts of HE on the microbiota of vaginal secretions in female animals need further exploration.

Several studies reported the effects of changes in sex hormones on metabolism. The adaptation to the external environment during HE causes significant alterations in nutrient absorption and metabolism (Coble et al., 2014), inducing abnormalities in energy (Lu et al., 2017), lipid (Victoria Sanz Fernandez et al., 2015), and amino acid (McCommis et al., 2015) metabolism in chickens, cows, and pigs. Exercise, diseases (Okamoto et al., 2018), and nutrition or drug intervention (Leng et al., 2019) affect the secretion of gonadal hormones, thus affecting serum metabolism. Unfortunately, the effects of heat-induced estrous cycle disorder (ECD) on metabolism in rats are less reported. Therefore, this study was performed to explore the pathogenesis of HE-induced ECD in female animals and lay the foundation for vaginal microbiota intervention and disease prediction. In our previous study, a stable model of estrous cycle disorder induced by high temperature had been built (An et al., 2020). Significant changes were observed in the estrus cycle in the heat exposure group compared with the control group. The prolongation of the estrus cycle is the specific index of the estrus cycle. Vaginal cytological smears were monitored at the same time each morning and evening during heat exposure to determine the estrous cycle of the animal. The rate of cumulative disorder of the estrous cycle in the heat exposure group (68.18%) was significantly higher than that of the control rats (13.63%) (*P* < 0.01). Significant extended changes were observed in the estrus cycle in the heat exposure group (4.95d ± 0.85d) compared with the control group (4.27d ± 0.21d) (*P* < 0.05). The vaginal secretions and serum were collected based on this model in this study, and it was used to characterize the differences from the globe in vaginal microbiota communities and serum metabolic profiles *via* 16S rDNA gene sequencing and metabolomics, respectively (An et al., 2020). This study was novel in evaluating the composition of vaginal microbiota and serum metabolite profiles of rats with ECD.

## Materials and Methods

### Animal Experiments

This study used the animal model established in a previous study (An et al., 2020). Pathogen-free, female Sprague–Dawley rats were obtained from Weitong Lihua Experimental Animal Technology Co., Ltd. (Beijing, China). The specific rats weighed 200 g ± 10 g. The next procedure was to house the rats in a room at a temperature of 23°C ± 1°C, relative humidity of 45%–60%, under a 12-h light-dark cycle, and access to food and water without limitation. The experimental animals were fed in cages for 2 days, followed by vaginal cytological smears at the same time each morning and evening. A total of 44 rats were randomly divided into two groups (with regular estrous cycles): (1) control group (C group)—the animals were fed under standard temperature and humidity conditions; and (2) HE group (H group)—each model animal was exposed to heat (38°C ± 0.5°C; relative humidity 55% ± 5%) for 2 h/day (9:00–11:00) in a small-animal heat chamber (Zheng et al., 2019; An et al., 2020). The experiment lasted for at least 90 days with free access to food and water. The blood samples of 10 rats were withdrawn from the abdominal aorta during diestrus in each group within 24 h after the last HE.

All procedures associated with animal care and use were carried out in strict accordance with the national institutes of health (NIH) guide for the care and use of laboratory animals (NIH Publications No. 8023, revised 1978). All the aforementioned procedures were approved by the ethics committee of the Institute of Environmental and Operational Medicine.

### Sample Collection and Preparation

The vaginal secretions of rats during diestrus in each group were taken within 24 h after the last HE. The aseptic cotton swabs were gently placed 1 cm–1.5 cm into the vagina of rats, rotated once, and removed. The cotton swabs were cut off aseptically, put into sterile conical tubes and then into liquid nitrogen for quick freezing, and stored at –80°C. During diestrus, the blood samples of rats in each group were obtained from the abdominal aorta. Sera were assessed by centrifuging the blood at 3,000 rpm with the endurance of 10 min, stored at –80°C, and used for nontargeted and targeted metabolomics analysis. Subsequently, these rats were euthanized. In addition, the blood samples and the vaginal secretions came from the same rat and were used for subsequent experimental analysis.

### 16S rDNA Gene Sequencing Analysis

#### Sequencing

The entire genomic DNA was separated by the previously described hexadecyl trimethyl ammonium bromide (CTAB)/sodium dodecyl sulfate (SDS) method (Ma et al., 2020). The consolidation and purity of DNA were monitored on 1% agarose gels. The DNA was diluted to 1 ng/μl with sterile water. The 16S rDNA gene was amplified using barcode-specific primers 515F-806R (V3–V4). The polymerase chain reaction (PCR) products were quantified and detected using 1× loading buffer (containing SYBR Green) and 2% agarose gel electrophoresis. An NEB Next Ultra DNA Library Prep Kit (Illumina; NEB, USA) was applied to generate a sequencing library, followed by the addition of index codes. The library quality was evaluated based on a Qubit @ 2.0 Fluorometer (Thermo Scientific, USA) and an Agilent BioAnalyzer 2100 system. Eventually, an Illumina MiSeq platform was used for the sequencing of the library, and fragments with paired-end reads of 300 bp were obtained.

### 16S rDNA Statistical Analysis

Fast length adjustment of short reads was used to merge paired-end reads outside the original DNA fragments. Sequence analysis was carried out with UPARSE-operational taxonomic units (OTUs) and UPARSE-OTUref algorithms using the UPARSE software package. Alpha (within samples) and beta (among samples) diversities were analyzed using in-house Perl scripts. Sequences with ≥ 97% similarity were assigned to the same OTUs. Additionally, the taxonomic classification information for each representative sequence was annotated by means of an RDP classifier. Quality control (QC) and OTU clustering of sequences were carried out using clustering analysis, principal component analysis (PCA), and Quantitative Insights Into Microbial Ecology Platform software and the information on the corresponding taxa of OTU (including class, phylum, order, genus, family, and species) and abundance was obtained. Three metrics, including Chao1, Simpson, and Shannon, were used to forecast the abundance, evenness, and diversity of species. Linear discriminant analysis effect size (LEfSe) was applied to perform the quantitative analysis of biomarkers in diversified groups. Analysis of similarities and multi-response permutation procedure based on different distance matrices of bray–curtis were used to detect the differences in microbiota between the two groups. The microbiota with significant differences between the two groups was screened (*P* < 0.05). NetworkX was targeted to discover the relationships between microbial communities (Peng et al., 2019) visualize them. The indexes of degree (DC), closeness (CC), and betweenness centrality (BC) were calculated and measured to characterize the topology highlights of constructed networks.

### Ultra-High-Performance Liquid Chromatography-Triple/Time-Of-Flight Mass Spectrometry (UHPLC-Q-TOF-MS/MS) Analysis of Serum Metabolomics

#### Nontargeted Metabolomics Sample Preparation and Analysis

A total of 100 μl thawed serum samples were mixed with 400 μl of precooled methanol–acetonitrile solution (1:1, v/v), placed in a vortex for 60 s, precipitated for 1 h at –20°C, and then centrifuged at 14,000 *g* and 4°C for 20 min. Finally, the supernatant was freeze-dried and then stored at –80°C for testing. The detection was carried out with an Agilent 1290 Infinity LC hydrophilic interaction liquid chromatography column. The column temperature was 25°C, the flow rate was 0.3 mL/min, and the mobile phase was composed of A (water + 25mM ammonium acetate + 25 mM ammonia) and B (acetonitrile). The linear change in gradient elution was as follows: 0–1 min, 95% B; 1–14 min, B from 95% to 65%; 14–16 min, B from 65% to 40%; 16–18 min, B 40%; 18–18.1 min, B from 40% to 95%; and 18.1–23 min, B maintained at 95%. The samples were consistently placed in an autosampler at 4°C during the entire analysis. The samples were continuously analyzed at random to reduce the fluctuations in instrument detection signals.

In the test, the QC samples were inserted to track and assess the stability of the test system and the accuracy of the experimental data. Electrospray ionization and positive and negative ion modes were adopted for detection. Ultra-high-performance liquid chromatography (UHPLC) and Triple TOF 5600 mass spectrometer (AB SCIEX, USA) were used to separate and analyze the samples.

The Electrospray ionization source conditions after hydrophilic interaction liquid chromatography chromatographic separation and the ionization conditions of secondary mass spectrometry accessed from information-dependent acquisition were determined, as described in a previous study (Liu et al., 2020).

### UHPLC-Q-TOF/MS Statistical Analysis

The original data were transformed into mzXML format with ProteoWizard (version 3.0.4146). Then, the XCMS program (http://xcmsonline.scripps.edu) was adopted for peak alignment, retention time correction, and peak area extraction. Structure identification of metabolites was performed by accurate mass matching (< 25 ppm) and secondary spectrum matching methods, and the laboratory database was searched.

The software SIMCA-P 14.1 (Umetrics, Umea, Sweden) was adopted to conduct multivariate analysis, including Pareto-scaled PCA and orthogonal partial least squares discriminant analysis (OPLS-DA). The sevenfold cross-validation and the response permutation testing were adopted to assess the firmness of the model. The variable importance in projection (VIP) value of each variable in the OPLS-DA model was measured and calculated to indicate its contribution to the classification. Metabolites with the VIP value >1 were further subjected to the Student *t*-test at the univariate level to determine the significance of each metabolite. The *P* values lower than 0.05 indicated a statistically significant difference.

#### Omics Association Analysis

The secondary metabolites screened by metabolomics were analyzed using Spearman correlation, and the bacterial flora with significant differences at the genus level was analyzed using 16S sequencing analysis. Further, the relationship between different bacterial flora and metabolites was obtained. R language and Cytoscape software were combined to analyze the matrix heat map, hierarchical clustering, association network, and so on. The suitable screening conditions were selected on the basis of calculations, and the final correlation and the network diagram between the final differential flora and metabolites were obtained.

### Targeted Metabolomics Analysis of Serum Neurotransmitters

#### Metabolite Extraction

For extracting metabolites, 100 μl of each serum sample was mixed with 400 μl of precooled pure acetonitrile containing 1% formic acid (FA) using a vortex. The protein was precipitated ultrasonically at –20°C for 1 h in an ice bath and centrifuged at 14,000 g and 4°C for 20 min. The supernatant was relocated and evaporated to dryness under a stream of nitrogen. During mass spectrometry detection, 100 μl of the mixture of (1% FA) ACN/water (1:1, v/v) was recombined and centrifuged at 14,000 *g* and 4°C for 20 min. Then, the sample was analyzed using the liquid chromatograph-mass spectrometer (LC-MS) system.

#### Chromatography–Mass Spectrometry

The samples were separated using an Agilent 1290 Infinity LC ultrahigh-performance liquid chromatography system (Agilent Technologies, CA, USA). Mobile phase: liquid A was 25 mM ammonium formate aqueous solution containing 0.1% FA, and liquid B was 0.1% FA acetonitrile. The sample was placed in an automatic sampler at 4°C. The column temperature was 45°C, and the flow rate was 300 μl/min. The corresponding liquid phase gradient of 2 min was performed as follows: liquid B changed linearly from 90% to 40%. A QC sample was set at specific intervals in the sample queue to identify and assess the stability and repeatability of the system.

A 5500 QTRAP mass spectrometer (AB SCIEX) was used for mass spectrometry analysis under a positive ion mode. The 5500 QTRAP ESI source conditions were as follows: source temperature, 450°C; ion source gas 1, 60; ion source gas 2, 60; curtain gas, 30; and ionic liquid voltage floating, 5000 V. The ion pair to be tested was detected by the MRM mode.

### Targeted Metabolomics Data Analysis

The chromatographic peak area and retention time were extracted using Multiquant software. The neurotransmitter criteria were used to correct retention time and identify metabolites.

## Results

### Variation In Vaginal Microbiota Composition In Rats With ECD

#### Structural Diversity of Vaginal Microbiota

**Figure 1:**
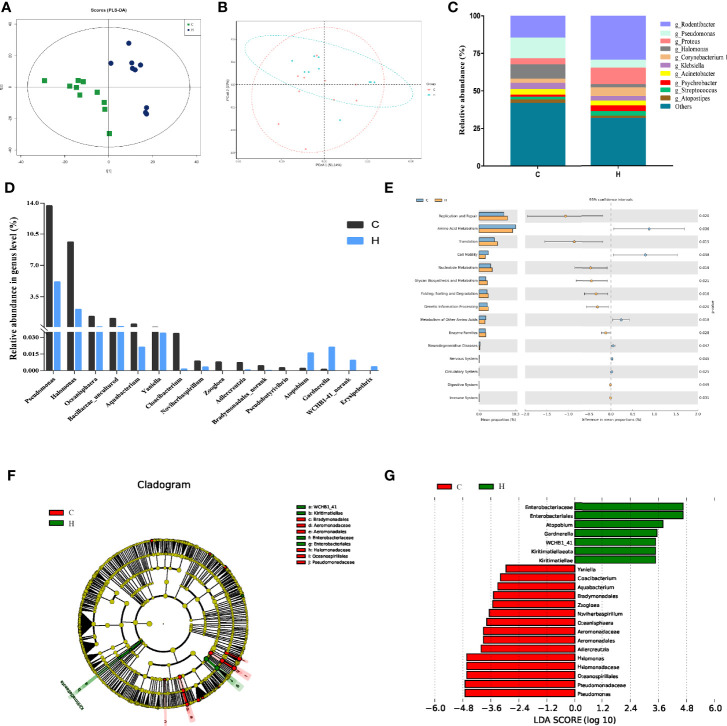
Vaginal microbial abundance and diversity in rats with HE-induced ECD. **(A)** The map of OTU abundance *via* PLS-DA analysis. **(B)** PCoA plot of weighted UniFrac distance of C and H group samples. The x-axis and the y-axis represent the first principal component and the second principal component respectively, and the percentage represents the contribution to the sample difference. **(C)** Top 10 most abundant genera in each group. **(D)** Distribution of vaginal bacteria with a significant difference in each group. **(E)** Predicted metabolic functions of vaginal microbiota in each group. KEGG pathways are shown in the extended error bar. The *P* value is shown on the right. **(F)** Phylogenetic distribution of vaginal microbiota from phyla to genera in each group *via* LEfSe analysis. **(G)** Cladogram generated from the LEfSe LDA analysis identifying the bacterial abundance between the two groups (LDA Core ≥ 2).

The characteristic spectra of vaginal secretions were evaluated using 16S rDNA gene sequencing to investigate the differences in the structural diversity of vaginal microbiota between rats with ECD and control rats. The data showed that 994,582 valid sequences were obtained from 20 samples in the two groups, and the average sample length was 255.97 bp. The partial least squares discriminant analysis (PLS-DA) analysis based on OTU abundance showed evident differences in microbial composition between the two groups (**Figure 1A**). The Chao, Simpson (Grabchak et al., 2017), and Shannon (Shannon, 1997) indices were used to estimate microbial richness and diversity and hence characterize microbial alpha-diversity (Whelan and Surette, 2017; Peng et al., 2019). The data showed that the Chao richness index was considerably higher in the H group than in the C group (*P* < 0.05) (**Table 1**). No difference was observed in Shannon and Simpson indices between the two groups. These results showed no difference in the microbial community’s diversity in the H group, but the colony abundance changed significantly (**Table 1**). Moreover, the beta-diversity of samples was analyzed using the principal coordinate analysis (PCoA) to characterize the similarity or difference in community composition between them. The distinct separations in the scatterplot indicated significant differences in community composition between the two groups, suggesting that HE obviously affected the vaginal microbiota (**Figure 1B**).

**Table 1:** Comparison of α diversity parameters between the control group and long-term heat exposure group.

Group	Chao	Shannon	Simpson
C	769.5 ± 159.29	3.50 ± 0.98	0.16 ± 0.13
H	938.83 ± 169.98*	4.45 ± 0.36	0.041 ± 0.02

C, control group; H, long-term heat exposure group. The values are presented as the mean ± standard deviation. Compared with the C group, *P < 0.05.

### Altered Composition of The Vaginal Microbiota in Rats With ECD

A total of 708 bacterial species were detected at the genus level. Among these, 606 and 561 species were detected in groups C and H, respectively. As shown in **Figure 1C**, the colony analysis of the relative abundance of the top 10 bacteria at the genus level showed that the abundance of the main dominant genera changed. For example, the relative abundance of *Pseudomonas*, *Halomonas*, and *Klebsiella* decreased from 13%, 9.61%, and 4.13% to 5.15%, 2.09%, and 3.15%, respectively. However, the relative abundance of *Rodentibacter, Proteus*, and *Corynebacterium* increased from 14.82%, 4.06%, and 2.71% to 29.67%, 11.07%, and 5.75%, respectively.

At the genus level, the Wilcoxon rank-sum test was used to analyze the differences in vaginal bacterial communities between the C and H groups. The results found that 16 genera were considerably different between the two groups (**Figure 1D**). The abundance of *Cloacibacterium*, *Aquabacterium*, *Bradymonadales_norank*, *Adlercreutzi*, *Oceanisphaera*, *Halomonas*, and *Pseudomonas* significantly reduced. Also, the abundance of *Gardnerella*, *WCHB1-41_norank*, *Erysipelothrix*, and *Atopobium* significantly increased.

The Kyoto Encyclopedia of Genes and Genomes (KEGG) pathways in 16S rDNA sequencing samples were predicted using PICRUSt software, and 42 functional pathways were found to be enriched. Among these, 15 pathways, including replication and repair, amino acid metabolism, cell motility, nucleotide metabolism, glycan biosynthesis, and metabolism of other amino acids, were significantly different, as shown in **Figure 1E** (*P* < 0.05). It revealed that the changes in vaginal microbiota in rats with HE-induced ECD might be related to the abnormal function of these pathways.

In general, LEfSe was used to determine any specific difference in bacterial group enrichment between them. As shown in the LEfSe phylogenetic distribution map, the significant differences in vaginal microbiota between the C and H groups were as follows: *Kiritimatiellae, WCHB1_41, Enterobacteriales, Enterobacteriaceae, Aeromonadales, Aeromonadaceae, Oceanospirillales*, and *Halomonadaceae* (**Figure 1F**). The results illustrated a remarkable difference in the distribution of vaginal microbiota between the two groups. The linear discriminant analysis (LDA) effect size distribution histogram of the two groups (**Figure 1G**) showed that 16 genera, including *Pseudomonas* and *Halomonas*, were the essential microorganisms in the C group, and 7 genera, including E*nterobacteriales*, *Gardnerella*, and *Atopobium*, were the crucial microorganisms in the H group (LDA Core ≥ 2).

### Correlation Network Analysis

**Figure 2:**
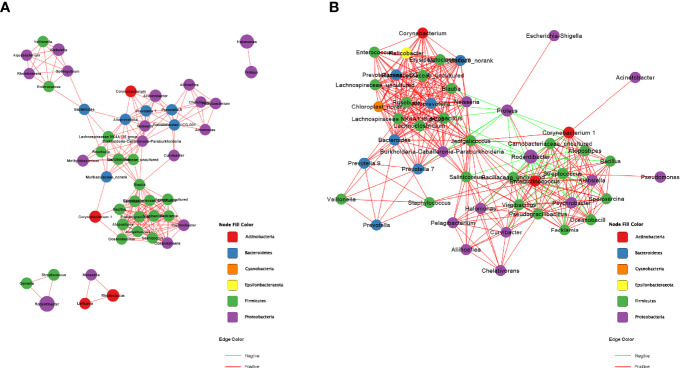
Correction network analysis of 50 most abundant genera in **(A)** normal rats and **(B)** ECD rats. The lines between nodes represent Spearman correction, and color intensity represents correlation coefficient. The red line means positive correlation and the green line means negative correlation. The color of the genus is based on the subordination of the gate, and the size shows the average relative abundance.

The correlation network analysis was performed at the genus level to decide whether HE was related to changes in the relevant structure and possible interaction structure of vaginal microbiota and also to identify possible keystone genera. **Figures 2A, B** represent the network diagrams of the top 50 genera with the highest vaginal abundance in group C and group H, respectively. The results showed that the network constructed had more edges (362 vs. 195) and a higher mean degree (14.48 vs. 7.8) in rats in the H group than in those in the C group, but the transitivity (0.77 vs. 0.82) was lower. These results indicated that the correlation was more significant in the H group than in the C group (**Figures 2A, B**). Additionally, DC, CC, and BC were employed to assess the significance of taxa within two networks. According to the high scores of these topological properties (DC > 0.1, CC > 0.2, and BC > 0.1), one genus *Jeotgalicoccus* was identified in the H group but no none in the C group.

### Alterations in the Serum Metabolic Profiles of Sample Rats With HE

#### Multivariate Statistical Analysis

**Figure 3:**
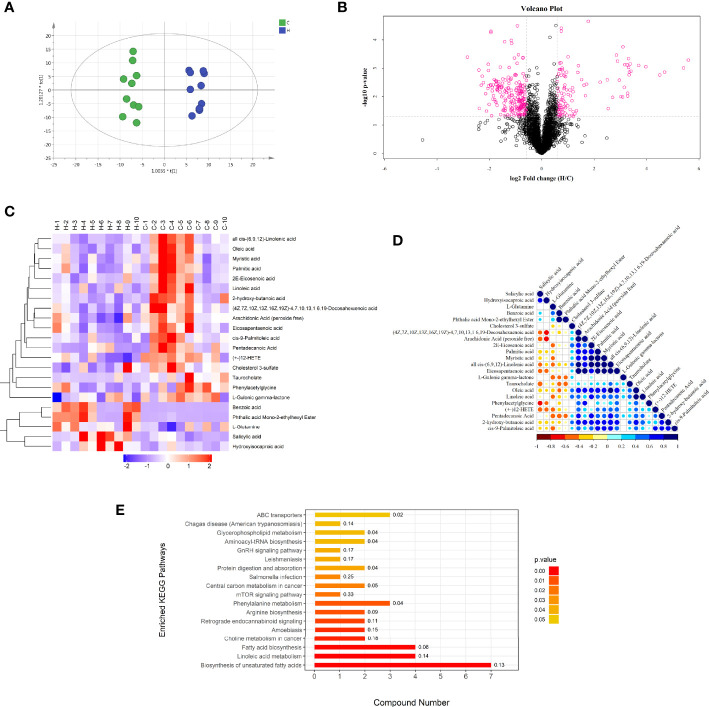
Negative ion mode multivariate statistical analysis, heat map, cluster analysis, and metabolic pathway. **(A)** OPLS-DA score chart of serum metabolite analysis between the C and H groups. t [1] is the predicted principal component to distinguish the variation between groups, and the orthogonal principal component t0 [1] reflects the variation within the group. **(B)** volcano map of differential metabolites between the C and H groups. The red spots in the map show metabolites with FC >1.5 and *P* value < 0.05. These metabolites were screened by univariate statistical analysis. **(C)** Results of hierarchical clustering of metabolites with apparent changes in serum samples. Red and blue represent higher and lower concentrations of metabolites, respectively (FC >1.5 and *P* value < 0.05). **(D)** correlation of metabolites with significant differences between the two groups. **(E)** Results of the KEGG pathway enrichment analysis of differential metabolites (*P* < 0.05). The X axis represents the number of significantly different metabolites were enriched in this pathway, and the value on the histogram is richFactor.

Liquid chromatography–mass spectrometry (LC/MS) detection in the two groups of serum samples showed 6243 ion peaks. An OPLS-DA model showed that the C and H groups had useful aggregation and obvious distinction between them, indicating that the model was anticipating and dependable, with significant differences in the abundance of serum metabolites between the two groups (**Figure 3A**) (R2Y = 0.973, Q2 = 0.746). The volcano diagram in **Figure 3B** shows the negative ion mode for different metabolites.

#### Metabolic Variation Analysis In Rats With ECD

The chosen endogenous metabolites were characterized according to the accurate mass and MS^E^ spectrum measurements obtained using Q-TOF/MS and compared with the data from the literature and/or online databases. The Supplementary Table shows the retention time, *m/z*, and VIP values.

The hierarchical clustering of each group of samples was carried out using the expression of metabolites with qualitatively significant differences. The results showed that the metabolic characteristics of the serum changed significantly due to the HE of rats in the H group. The tendencies of variation of these metabolites were depicted with a heat map (**Figure 3C**). A total of 22 potential metabolic biomarkers (VIP > 1.0; *P* < 0. 05) were screened (**Table 2**). Among these, the relative concentrations of 9 metabolites in the serum of rats significantly increased after HE, while 13 metabolites significantly decreased. The metabolite correlation analysis found that these metabolites interacted with each other (**Figure 3D**), including a negative correlation between oleic acid and benzoic acid. Significant metabolic pathway changes occurred in rats with ECD, including lipid metabolism, linoleic acid metabolism, amino acid metabolism, mammalian target of rapamycin (mTOR) signaling pathway, GnRH signaling pathway, and other metabolic pathways (*P* < 0.05) (**Figure 3E**).

**Table 2:** Serum metabolites significantly changed in the control group and the long-term heat exposure group.

Name	Adduct	Description	VIP	Fold change	*p*-value
M121T210	(M-H)-	Benzoic acid	18.838	48.256	0.001
M277T74	(M-H)-	Phthalic acid Mono-2-ethylhexyl Ester	7.742	12.971	0.001
M167T65	(M+H)+	1,2-Benzenedicarboxylic acid	1.232	8.290	0.001
M131T236	(M-H)-	Hydroxyisocaproic acid	2.103	2.293	0.034
M137T62_2	(M-H)-	Salicylic acid	1.611	2.099	0.006
M175T1053	(M+H)+	L-Arginine	3.826	2.014	0.001
M759T297	(M+Na)+	Thioetheramide-PC	3.862	1.446	0.033
M118T549	(M+H)+	Betaine	2.851	1.263	0.018
M145T691	(M-H)-	L-Glutamine	1.580	1.245	0.039
M303T74	(M-H)-	Arachidonic Acid (peroxide free)	9.163	0.754	0.022
M177T253	(M-H)-	L-Gulonic gamma-lactone	1.476	0.738	0.031
M301T76	(M-H)-	Eicosapentaenoic acid	1.469	0.708	0.045
M241T81	(M-H)-	Pentadecanoic Acid	2.344	0.646	0.020
M327T74	(M-H)-	(4Z,7Z,10Z,13Z,16Z,19Z)-4,7,10,13,1 6,19-Docosahexaenoic acid	6.393	0.571	0.004
M103T342	(M-H)-	2-hydroxy-butanoic acid	1.884	0.539	0.005
M253T254	(M-H)-	cis-9-Palmitoleic acid	1.518	0.537	0.036
M277T79	(M-H)-	all cis-(6,9,12)-Linolenic acid	4.804	0.512	0.006
M281T188	(M-H)-	Oleic acid	2.230	0.509	0.003
M192T320	(M-H)-	Phenylacetylglycine	1.442	0.499	0.040
M319T84	(M-H)-	(+-)12-hydroxyeicosatetraenoic acid	9.645	0.496	0.002
M194T362	(M+H)+	Phenylacetylglycine	1.020	0.495	0.028
M309T74_2	(2M+H)+	Lavandulol	2.002	0.350	0.039

VIP, variable importance in the projection.

### Correlation Analysis of Vaginal Microbiota and Serum Metabolic Phenotype

**Figure 4:**
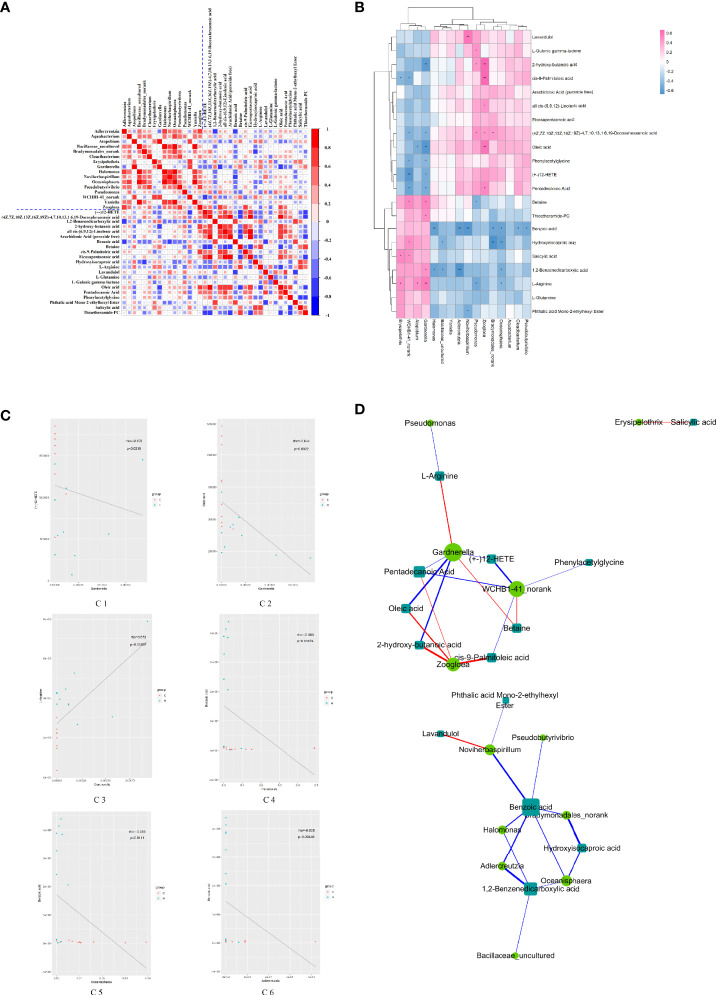
Relationship between vaginal microorganisms and serum metabolites. **(A)** Correlation coefficient matrix thermograph illustrating the functional correlation between perturbed vaginal microbiota and altered serum levels of metabolites. With the blue dotted line in the picture as the dividing line, it is divided into four quadrants. The upper left corner shows the correlation between the significantly different Vaginal flora, and the lower right corner shows the correlation between the significantly different metabolites. Both the upper right corner and the lower left corner show the correlation between the significantly different flora and metabolites (mirror image symmetry). The correlation coefficient r is expressed by color, r > 0 means positive correlation (red), and r < 0 means negative correlation (blue). The darker the color, the stronger the correlation. **(B)** Heat map summarizing the correlation between perturbed vaginal microbiota and altered serum levels of metabolites. *P value < 0.05, **P value < 0.01. **(C)** Scatter plots showing a statistical correlation between the relative abundance of altered vaginal bacteria and the mass spectrum intensities of some typical serum metabolites. (D1) Between *Gardnerella* and (+-) 12-HETE, (D2) between *Gardnerella* and oleic acid, (D3) between *Gardnerella* and L-arginine, (D4) between *Halomonas* and benzoic acid, (D5) between *Oceanisphaera* and benzoic acid, and (D6) between *Adlercreulzia* and benzoic acid. In the graph, the scattered dots represent the samples, and the colors correspond to different groupings. Rho is the Spearman correlation coefficient between the relative microbiota abundance and the metabolite intensity. The *P* value is the significant level of the rho. In **(A–C)**, blue represents negative correlation (r < 0), and red represents positive correlation (r > 0); the darker the color, the stronger the correlation. **(D)** Network diagram of the correlation between vaginal microbiota and serum levels of metabolites. The microbiota and metabolites with absolute correlation coefficient [0.3, 1] were analyzed by the Spearman correlation network. The circle represents the altered bacteria, and the rectangle represents the altered serum levels of metabolites. The thickness of the line is proportional to the absolute value of the correlation coefficient. The node size positively correlates with its degree, that is, the greater the degree, the larger the node size.

The correlation coefficient matrix thermograph was generated *via* spearman’s rank-order correlation analysis to determine the potential correlation between altered vaginal microbiota and serum potential metabolite biomarkers. The results showed a significant correlation between the alterations in vaginal microbial composition and serum metabolic spectrum after HE (| *r* | > 0.5; *P* < 0.05) (**Figure 4A**). As shown in **Figure 4B**, 52 pairs of significant microbiota–metabolite correlations were determined, including 21 pairs of significant positive correlation and 31 pairs of significant negative correlation. For example, the relative abundance of *Pseudobutyrivibrio*, *Oceanisphaera*, *Bradymonadales-norank*, *Noviherbaspirillum*, *Adlercreutzia*, and *Halomonas* negatively correlated with the concentrations of pentadecanoic acid, betaine, benzoic acid, 1,2-benzenedicarboxylic acid, L-arginine, and phthalic acid mono-2-ethylhexyl ester (*P* < 0.05). The relative abundance of *Zoogloea* and *Pseudomonas* positively correlated with the concentrations of oleic acid, 2-hydroxy-butanoic acid, and *cis*-9-palmitoleic acid (*P* < 0.05). **Figure 4C** shows several typical vaginal flora–related metabolites associated with specific vaginal bacteria. For example, in rats with HE, the concentration of benzoic acid negatively correlated with the relative abundance of *Halomonas, Oceanisphaera, Adlercreutzi*a. and **Figure 4D** reflects the correlation between significantly different microbiota and significantly different metabolites. Notably, the *Gardnerella* node of the dominant bacteria in the vaginal flora was the largest in the network diagram, with a positive correlation with the concentrations of L-arginine and betaine and a negative correlation with the concentrations of many metabolites such as oleic acid, 2-hydroxy-butanoic acid, and pentadecanoic acid, suggesting that the changes in the concentrations of these metabolites might be caused by the changes in *Gardnerella*. In addition, the metabolite benzoic acid node was the largest in the network map, with a negative correlation with the abundance of many genera such as *Adlercreutzia*, *Halomonas*, and *Noviherbaspirillum*, suggesting that the change in the concentration of benzoic acid might affect the change in the abundance of these florae. The concentration of oleic acid decreased by 0.51 times, with a negative correlation with the abundance of *Gardnerella*, but a positive correlation with *Zoogloea*. The aforementioned correlation data implied that the sample rats with ECD presented considerable taxonomic perturbations in the microbiome, which might lead to a significantly altered metabolomics profile.

### Effects of HE on the Serum Levels of Neurotransmitters

**Figure 5:**
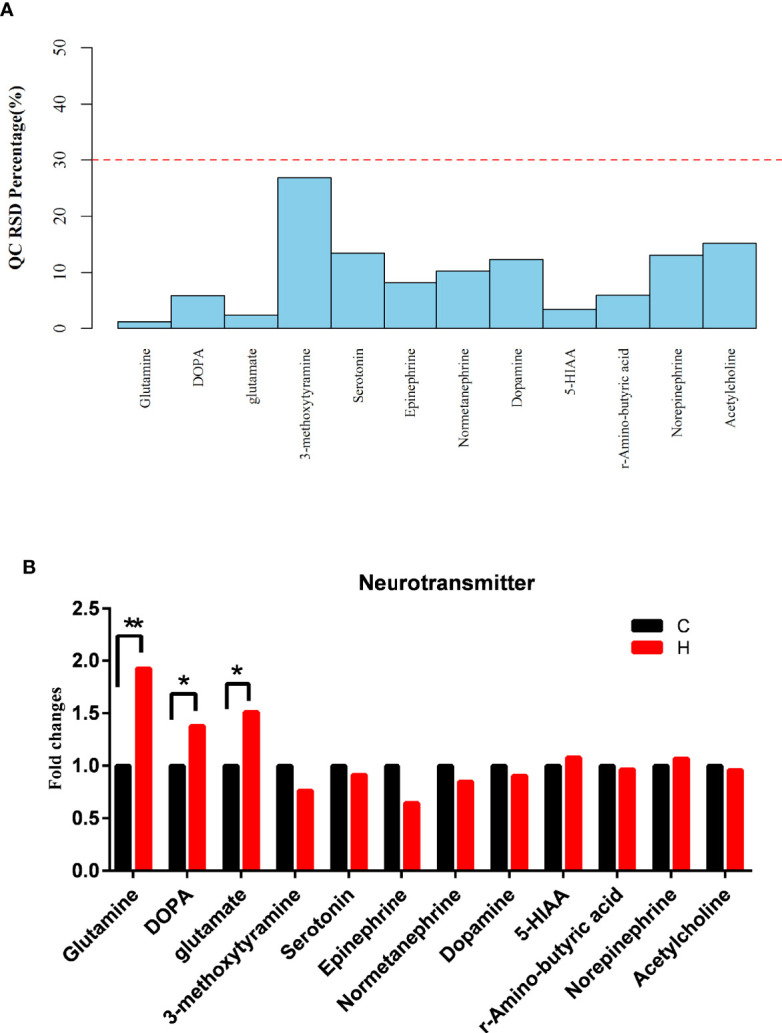
Effects of HE on serum neurotransmitters. **(A)** RSD distribution of QC samples of serum neurotransmitters. **(B)** Changes in the levels of serum neurotransmitters, as detected by targeted metabolomics analysis. Compared with the C group, **P < 0.01, *P < 0.05. (DOPA represents L-DOPA in the above two graphs).

Targeted metabolomics was used to observe the effect of HE on the levels of serum neurotransmitters. The neurotransmitters with relative standard deviations (RSD) less than 30% indicated that the data in the sample were steady and accurate (**Figure 5A**). A total of 12 neurotransmitters were detected in serum samples, of which 3 changed significantly, including glutamine, glutamate, and L-3,4-dihydroxyphenylalanine (L-DOPA), which increased by 1.93-fold, 1.51-fold, and 1.38-fold, respectively (**Figure 5B**). The previous results and data suggested that the levels of glutamatergic neurotransmitters and monoaminergic neurotransmitters changed significantly in ECD. Also, an increase in the glutamine level in serum metabolomics was observed. Hence, the results suggested that the changes in vaginal microorganisms might be closely related to glutamate metabolism.

## Discussion

HE affects the reproductive function, posing a severe threat to animal husbandry, human beings, and other mammals. However, relevant mechanisms have not been fully elucidated. Also, the mechanism of HE-induced ECD is rarely reported. Emerging studies support the relationship between the microbiota, health, and disease. Hence, the regulation of vaginal microbiota may be developed into a methodical approach to detect the HE-induced damage to reproductive function. Previous studies revealed that the alterations in vaginal microbiota affected the level of autophagy of vaginal epithelial cells (Nasioudis et al., 2017) and induced BV (Lewis et al., 2013) and local vaginal inflammation in pregnant mice (Sierra et al., 2018). The changes in maternal vaginal microbiota also affected the healthy brain nerve development and intestinal microbiota colonization of offspring (Jasarevic et al., 2018). Thus, understanding the microbiota composition of rats with HE-induced ECD may help formulate microbial intervention strategies. Recent studies have focused on the effects of HE on the intestinal and fecal microbiota of economic animals, such as chickens, pigs, cows, buffalos, and so on (Chen et al., 2018; He et al., 2019; Shi et al., 2019; Zhu et al., 2019). They have promoted a comprehensive analysis of the vaginal microbiota of rats with HE-induced ECD.

Therefore, 16S rDNA gene sequencing was performed to investigate the effect of ECD on vaginal microbiota. In this study, significant changes in microbiota at both phylum and genus levels were observed in the vaginal secretion of rats with HE-induced ECD. The colony abundance altered significantly in the H group compared with the C group. At the genus level, the abundance of 12 and 4 genera was remarkably higher and lower in vaginal secretions, respectively (**Figure 1D**). Among these bacteria with low relative abundance, the abundance of *Cloacibacterium*, *Adlercreutzi*, and *Oceanisphaera* were closely related to reproduction and neuroendocrine function. *Cloacibacterium* is the dominant bacteria in the cervix, endometrium (Yeoman et al., 2013), and vagina (Winters et al., 2019). Combined with *Acinetobacter and Pseudomonas*, these taxa may be common members of microbial homeostasis in the lower reproductive tract (vagina) and upper reproductive tract (endometrium). The aforementioned taxa are stable in the endometrium, while cervicectomy leads to a decrease in *Cloacibacterium* (Winters et al., 2019). A decrease in the abundance of vaginal *Cloacibacterium* caused by HE affects the homeostasis of endometrial microorganisms. The relative abundance of intestinal *Adlercreutzia* was affected by temperature (increases after cold exposure in mice), producing estriol, an estrogen with remarkable antioxidant activity (Zietak et al., 2016). Estriol strongly suppressed GnRH-induced luteinizing hormone (LH) secretion (Otsuka and Kadokawa, 2017), and its relative abundance negatively correlates with anxiety (Xu et al., 2019). LH and anxiety are closely related to ECD. *Oceanisphaera* can cause a series of extravaginal infections (Huys, 2014). Of note, *Pseudomonas* and *Halomonas* are conditional pathogens involved in body immunity. *Pseudomonas* can cause the release of a series of virulence-related substances, comprising endotoxin, elastase, and so forth, which are closely related to a bacterial infection and the development of pelvic inflammation in women (Vander and Prabha, 2019). *Halomonas* is active and pathogenic in the feces of heat-stressed pigs (He et al., 2019). Most of these bacteria are Gram-negative aerobic bacteria and also conditional pathogens. Most of the aforementioned microorganisms are related to inflammatory and lipid metabolic pathways. HE-induced ECD may be related to the reduction in the abundance of these microorganisms involved in inflammatory reaction and lipid metabolism.

*Gardnerella* and *Atopobium* are most closely related to BV. The increase in the number of bacteria significantly increases the risk of BV (Mastromarino et al., 2002). Previous studies indicated that the aforementioned genera were involved in the formation of BV biofilms; up to 10%–15% of patients with BV failed to respond to initial antimicrobial therapy (Oduyebo et al., 2009; Menard, 2011; Machado and Cerca, 2015), accompanied by an increase in the level of inflammatory mediators (Castro et al., 2020). In the present study, the abundance of *Gardnerella* and *Atobacter* increased, indicating that HE-induced ECD might increase the occurrence of diseases such as BV, or the inflammatory response caused by flora imbalance might induce the formation of ECD. These genera might have a relatively less impact on the body due to their low abundance. In summary, the results showed that the intricate balance between gram-negative aerobes and gram-positive anaerobes in vaginal microecology was broken after HE. These bacterial alterations led to abnormal inflammatory and immune responses and increased the incidence of ECD and vaginal infectious diseases.

Furthermore, the 16S rDNA results indicated that HE had less effect on the vaginal microbiota, with no drastic or subversive changes. It was speculated that the body had dynamic alterations in stress and adaptation during HE, and the adaptive response mitigated the effect of HE on the microecology of the vaginal microbiota. Consistently, the vagina was relatively closed; the effect of conditionally pathogenic bacteria was inhibited without sexual disturbance to the vaginal microbiota (Vodstrcil et al., 2017). Hence, the impact of HE on the estrous cycle was not as significant as that of gynecological diseases, and the effect on the vaginal microbiota of unmarried or unmated female rats was relatively less.

Recently, numerous studies showed that metabolic changes were paralleled by intestinal microbiota disorder during the development of diseases. Similarly, the metabolic changes were paralleled by vaginal microbiota disorders during the occurrence and development of impaired reproductive function; BV (Vitali et al., 2015); fungal, yeast, and vulvovaginal candidiasis infections (Parolin et al., 2015); preterm birth (Hill et al., 2017); urinary tract infections (Chorna et al., 2020); and other diseases. Serum metabolome characterization can promote the understanding of the microbiota response to the perturbations of the vaginal microbiota.

The present study showed that the levels of 9 metabolites were remarkably higher and those of and 13 metabolites were lower in rats in the H group (**Table 2**). Among the metabolites with increased levels, benzoic acid had antibacterial activity, which can increase the abundance of *Lactobacillus* and *Bacillus* in the intestinal tract, and also promote the growth performance of suckling and weaning pigs (Kluge et al., 2006; Mao et al., 2019). As an organic acid and a substitute for antibiotics, it can promote other antibacterial substances (such as thymol) to enter bacteria and play an antibacterial role (Diao et al., 2015). Hydroxyisocaproic acid has antibacterial activities and an immunomodulatory effect (Nieminen et al., 2014); it is a protein fermentation product formed by the lactic acid bacteria in the vagina *via* the leucine degradation pathway (Sakko et al., 2012). HE can induce an inflammatory response and ECD in rats (An et al., 2020). The increased level of hydroxyisocaproic acid might be the pathway for the self-regulation of HE-induced ECD in female rats. As a conditionally essential amino acid, L-arginine is the precursor of nitric oxide synthesis. Nitric oxide participates in reproduction and affects the estrous cycle (Sica et al., 2009). In the present study, the expression of L-arginine was upregulated, indicating that it might be related to the formation and regulation of ECD. Studies have shown that L-arginine participates in sugar and fatty acid metabolism, protein metabolism, and synthesis of various amino acids (Monti et al., 2017). During HE, L-arginine can improve intestinal mucosal barrier function by activating the adenosine 5’-monophosphate-activated protein kinase pathway (Xia et al., 2019) and block the apoptosis pathway related to acute stress such as heat injury (Chatterjee et al., 2005). Therefore, it is suggested that L-arginine might reduce the disorder of the estrous cycle in rats by regulating glucose and lipid metabolism and amino acid synthesis during HE. Studies have shown that high temperature inhibits ovarian function and reduces the follicular growth rate (Zheng et al., 2019). As a potential endocrine disruptor with estrogenic activity, the phthalic acid mono-2-ethylhexyl ester may inhibit follicular growth through the oxidative stress pathway (Zhang et al., 2018) and by reducing estradiol production (Wang et al., 2012). Therefore, the increased expression of phthalic acid mono-2-ethylhexyl ester in serum after HE may lead to the disorder of rat estrous cycle and affect the reproductive health by inhibiting follicular growth in rats.

Among the metabolites with decreased levels, lavandulol has an antimicrobial effect on bacteria and a significant antioxidant effect on multifarious microorganisms (Arantes et al., 2016). The mechanism of action consists of disturbing the lipid structure of cell membranes, thereby leading to cell death (Bialon et al., 2019). Phenylacetylglycine is the metabolite of essential amino acid phenylalanine (Kristiansen et al., 2014). The decreased concentration of phenylalanine indicates abnormal phenylalanine concentration and abnormal glucose and lipid metabolism (Kim et al., 2016). Oleic acid participates in the change in the estrous cycle. It affects the sexual arousal and reproductive behavior of bulls (Muniasamy et al., 2017). It has carryover effects interfering with GnRH-induced calcium mobilization in pituitary gonadotropes, thereby affecting LH release (Salehi et al., 2015). Further, *cis*-(6, 9, 12)-linolenic acid, arachidonic acid, and 12-eicosapentaenoic acid are fatty acids. Also, *cis*-(6,9,12)-linolenic acid is an intermediate product of linoleic acid metabolism, which can be converted into arachidonic acid and has anti-inflammatory effects. **(+-)**12-Hydroxyeicosatetraenoic acid (**(+-)**12-HETE) is a metabolite of arachidonic acid. These three metabolites have pro-inflammatory and lipid-promoting effects (Abdullah et al., 2017) or participate in pro-inflammatory and oxidative stress (Cheng et al., 2019). Previous studies showed that the level of arachidonic acid decreased significantly in myocardial ischemia–induced heat stress (van der Vusse et al., 1998). Eicosapentaenoic acid is an omega-3 polyunsaturated fatty acid that can promote beneficial effects such as anti-inflammation, vasodilation, and anti-aggregation (Nelson et al., 2017). The ratio of arachidonic acid to eicosapentaenoic acid is an essential indicator of cellular inflammation (Tesei et al., 2017). Simultaneously, remarkable alterations in serum metabolism in rats with ECD might be related to oxidative stress and lipid metabolism. Among these, the formation of oleic acid and ECD were most closely correlated.

Furthermore, the metabolic pathway enrichment analysis identified a change in different metabolic pathways, such as biosynthesis of unsaturated fatty acids, linoleic acid metabolism, GnRH signaling pathway, mTOR signaling pathway, phenylalanine metabolism, and arginine biosynthesis. The previous proteomic and gene sequencing results also showed that HE might affect immune-related signaling pathways and carbohydrate/lipoprotein metabolism–related signaling pathways (Wang et al., 2016; Fan et al., 2019). The results suggested that the alterations in the levels of different metabolites might lead to ECD during HE and affect the health of the endocrine, immune, and reproductive systems of rats through the lipid metabolism pathway, amino acid metabolism pathway, mTOR signaling pathway, and GnRH signaling pathway.

Spearman correlation analysis was performed to explore the correlation between the altered vaginal genera and the levels of serum metabolites. Significant relationships were observed between vaginal microorganisms and the levels of serum metabolites involved in energy metabolism, immunity, and inflammation, indicating that the disorder of vaginal microbiota was related to the change in the metabolic phenotype. Of particular interest, the relative abundance of *Gardnerella* correlated negatively with the concentrations of both **(+-)**12-HETE and oleic acid and positively with the concentration of L-arginine. **(+-)**12-HETE and oleic acid could control the inflammatory response and lipid metabolism, indicating that vaginal microbiota imbalance might affect host immunity and energy metabolism. L-Arginine participates in many metabolic pathways, such as sugar and fatty acid metabolism, and the synthesis of various amino acids, indicating that the imbalance of vaginal microbiota might affect host energy metabolism and amino acid metabolism. Moreover, the abundance of *Pseudomonas* negatively correlated with the concentration of L-arginine, indicating that vaginal microbiota alterations were related to glucose and lipid metabolism. Further, a negative correlation was found between the relative abundance of *Adlercreutzia*, *Oceanisphaera*, and *Halomonas* and the concentration of benzoic acid, and between the abundance of *Oceanisphaera* and the concentration of hydroxyisocaproic acid. Benzoic acid and hydroxyisocaproic acid have antibacterial activity, and their correlation further indicates that the alterations in the abundance of conditional pathogens are related to the concentrations of benzoic acid and hydroxyisocaproic acid. These results hinted that the structural changes in vaginal microorganisms in rats with ECD were closely related to the metabolic phenotype of the host.

The detection of serum neurotransmitters is a kind of targeted metabolite detection, including acetylcholine, serotonin, histamine, and so on. The results of vaginal microbiome and serum metabolomics were used to analyze the effect of vaginal microorganisms on the host. The levels of 12 neurotransmitters were detected in the serum. The results suggested that the serum levels of neurotransmitters changed after HE (**Figure 5B**). The levels of glutamate, glutamine, and L-DOPA increased significantly. Glutamate was an excitatory neurotransmitter that could control hormone release and lipid metabolism and could be metabolized to glutamine through the tricarboxylic acid cycle (Wu, 2009). The reports also confirmed a strong correlation between the increase in glutamine and glutamate metabolism and the increase in the reactive oxygen species level. That is, glutamine and glutamate had an antioxidant effect. The results above suggested that the level of oxidative stress in the body increased, and glutamine and glutamate might be related to the development of HE-induced ECD in rats. Of particular interest, the serum level of L-DOPA, which is the precursor of dopamine, significantly increased; L-DOPA is closely related to cognition and neuroendocrine function and can pass through the blood-brain barrier (Lerner et al., 2017; Gonzalez-Carter et al., 2019). The finding indicated that the changes in the vaginal microbiome in HE-induced ECD rats could affect the host through serum neurotransmitters.

Our results about potential microorganisms and metabolites are limited to explanation of the molecular mechanisms associated with ECD. The influence of vaginal microbiota and metabolites may be part of the most extensive and multifactorial process, which requires further exploration. Microorganisms and metabolites not significantly related to ECD are still valuable and worthy of further investigation. Future studies should perform the functional verification of candidate bacteria and explore the mechanism of their effects on the host, thus laying an experimental foundation for predicting physiological cycle disorders caused by high temperature and proposing intervention measures.

## Conclusions

In summary, HE disturbed the abundance of vaginal microbiota and its metabolic curve with serum metabolites. The changes in the abundance of vaginal microorganisms, such as *Gardnerella, Pseudomonas*, and *Adlercreutzia*, and the concentrations of serum metabolites oleic acid, L-arginine, and benzoic acid might affect the estrous cycle, embryonic development, and reproductive inflammation of female rats by interfering with lipid metabolism, amino acid metabolism, mTOR signaling pathway, GnRH signaling pathway, and so on. Hence, they might serve as early monitoring markers for HE-induced ECD. The findings of this study provided a new insight for further investigating the mechanism of health effects of long-term HE on the reproductive system of female rats.

## Data Availability Statement

The original contributions presented in the study are publicly available. This data can be found here: https://www.ncbi.nlm.nih.gov/bioproject/PRJNA658610.

## Ethics Statement

All procedures associated with animal care and use were carried out in firm and rigid accordance with the National Institutes of Health Guide for the Care and Use of Laboratory Animals (NIH Publications No.8023, revised 1978). All the procedures mentioned above were approved by the ethics committee of the Institute of Environmental and Operational Medicine.

## Author Contributions

GA, JW and DY conceived, designed, and supervised the project. GA, LZ and YZ executed the project. LF performed statistical analysis and visualization. JC, MW, XC and CL performed investigation and resources. LF and GA wrote the original manuscript. JW and DY reviewed the final manuscript. JW and DY provided funding acquisition. All authors contributed to the article and approved the submitted version.

## Funding

This study was supported by the grants of Tianjin Institute of Environmental and Operational Medicine (BWS17J025).

## Conflict of Interest

The authors declare that the research was conducted in the absence of any commercial or financial relationships that could be construed as a potential conflict of interest.

## Abbreviations

HE, Long-term heat exposure; ECD, estrous cycle disorder; 16S rDNA, 16S ribosomal deoxyribonucleic acid; BV, bacterial vaginosis; mTOR, mammalian target of rapamycin; GnRH, gonadotropin-releasing hormone; C group, control group; H group, Long-term heat exposure group; OTUs, operational taxonomic units; QC, quality control; PCA, principal component analysis; DC, indexes of degree; CC, closeness; BC, betweenness centrality; UHPLC-Q-TOF-MS/MS, ultra-high-performance liquid chromatography-triple/time-of-flight mass spectrometry; OPLS-DA, orthogonal partial least-squares discriminant analysis; LDA, linear discriminant analysis; LEfSe, linear discriminant analysis effect size; PLS-DA, partial least squares discriminant analysis; FA, formic acid; VIP, variable importance in the projection; PCoA, principal coordinate analysis; KEGG, Kyoto Encyclopedia of Genes and Genomes; LDA, linear discriminant analysis; LC/MS, liqid chromatography-mass spectrometry; LH, luteinizing hormone; RSD, relative standard deviation; L-DOPA, L-3,4-Dihydroxyphenylalanine; **(+-)**12-HETE, **(+-)**12-hydroxyeicosatetraenoic acid.
